# Development of a novel pseudovirus-based quality control material for HIV-1 nucleic acid testing and its application in external quality assessment

**DOI:** 10.1128/spectrum.00269-25

**Published:** 2025-06-10

**Authors:** Di Han, Xin Zhang, Mingzhu Niu, Pinliang Pan, Wenge Xing, JingDong Song, Cong Jin

**Affiliations:** 1 National Key Laboratory of Intelligent Tracking and Forecasting for Infectious Diseases, National Center for AIDS/STD Control and Prevention, Chinese Center for Disease Control and Prevention 12415, Beijing, China; 2 Department of Epidemiology, School of Public Health, China Medical University 540411 , Shenyang, China; 3 National Institute of Pathogen Biology, Chinese Academy of Medical Sciences and Peking Union Medical College 220736, Beijing, China; Wannan Medical College, Wuhu, Anhui, China

**Keywords:** pseudovirus, lentiviral vector, HIV-1 nucleic acid testing, quality control material, external quality assessment

## Abstract

With the widespread application of HIV-1 nucleic acid testing (NAT) in China, particularly in the diagnosis of HIV-1 infection, ensuring the accuracy of NAT results through quality control has become critically important. However, existing HIV-1 NAT quality control materials (QCMs), such as clinical plasma samples and inactivated HIV-1 cell culture supernatants, have limitations in sustainability, biosafety risks, and costs. MS2-armed RNA (MS2) does not replicate the biological characteristics of natural viruses or the complexities of the extraction and detection processes associated with authentic viral particles. To address these limitations, this study developed a novel HIV-1 NAT QCM based on HIV-1 pseudovirus (PsV). HIV-1 PsV packaged using an improved four-plasmid lentiviral vector (LV) system could be generated with a high concentration of up to 10⁹ copies/mL. The HIV-1 PsV mimics the morphology of the real virus and is capable of only single-cycle infection, thereby ensuring biosafety. The HIV-1 PsV-based QCM demonstrated excellent homogeneity, absence of matrix effects, stability for 7 days at 4°C and −20°C, and the ability to withstand up to five freeze-thaw times. We further found that HIV-1 PsV outperformed inactivated HIV-1 and MS2 in terms of short-term stability and freeze-thaw stability, respectively. Additionally, the PsV-based QCM was successfully detected by 12 commercial HIV-1 NAT quantification kits in the Chinese market and demonstrated excellent performance in an external quality assessment (EQA) involving 60 laboratories. In summary, the novel HIV-1 PsV-based QCM can serve as a safe and sustainable alternative to existing HIV-1 NAT QCMs for EQA of HIV-1 NAT laboratories.

**IMPORTANCE:**

This study proposes a novel strategy to prepare HIV-1 nucleic acid testing (NAT) quality control material (QCM) using HIV-1 pseudovirus (PsV) packaged by an improved four-plasmid lentiviral vector (LV) system. The HIV-1 PsV-based QCM can simulate authentic virus particles and better monitor the entire HIV-1 NAT process, including nucleic acid extraction, amplification, and detection. The innovative HIV-1 NAT QCM possesses several desirable characteristics: biosafety, homogeneity, stability, and the ability to be prepared at high concentrations and on a large scale, significantly reducing production costs. Compared to commonly used QCMs such as inactivated HIV-1 and MS2, the HIV-1 PsV demonstrates superior stability and better meets the requirements for transportation, storage, and quality control applications of HIV-1 NAT laboratory. Particularly, the ability of HIV-1 PsV to accommodate the insertion of large nucleic acid sequences provides a solid technical foundation for developing more advanced quality control solutions in the future.

## INTRODUCTION

HIV-1 nucleic acid testing (NAT) plays an irreplaceable role in monitoring the effectiveness of antiviral therapy and in the diagnosis of infection. Compared with HIV serological tests, HIV NATs offer advantages such as greater sensitivity and specificity (1). More importantly, the shorter testing window reduces missed the detection of early acute HIV infection, which is critical for preventing HIV infection transmission (2). In 2015, the national guidelines for the detection of HIV/AIDS were revised in China and for the first time recommended NAT as a supplemental assay for the confirmatory tests (3). By the end of 2024, more than 800 laboratories across China’s health institutions were capable of performing HIV-1 NAT, according to internal data from China CDC. The geographical distribution of HIV NAT laboratories has progressively extended from provincial-level to prefecture-level, and in regions with a high prevalence of HIV, it has even extended to some county-level areas (4). Although the accessibility of HIV-1 NAT is increasing in China, it is crucial to implement strict laboratory quality control to ensure the accuracy of test results (4).

The accuracy of HIV NAT results is influenced by multiple factors, including sample quality, operator proficiency, instruments, and reagents, highlighting the indispensable role of performing quality control. Quality control in NAT primarily relies on the utilization of NAT quality control material (QCM). Currently, HIV-1 NAT QCMs are mostly prepared from clinical plasma samples, inactivated HIV-1 cell culture supernatant, or MS2-armed RNA (MS2) (5, 6). Clinical samples have long been considered the gold standard for QCMs (7, 8). However, it is difficult to obtain clinical samples from HIV-1-infected individuals in large quantities on a sustainable basis, making these samples unable to meet the growing demand for quality control (4). Inactivated HIV-1, which is typically sourced from high biosafety level laboratories, presents practical challenges due to stringent requirements for laboratory facilities and personnel, as well as costs, further limiting its feasibility for widespread use. Furthermore, both clinical samples and inactivated HIV-1 carry inherent infection risks, necessitating inactivation procedures during the preparation of QCMs. Although MS2 is a safer and more stable alternative (9), it does not replicate the biological characteristics of natural viruses or the complexities of the extraction and detection processes associated with authentic viral particles.

The HIV-1 lentiviral vector (LV) system offers a promising new approach by producing HIV-1 pseudovirus (PsV) that more accurately simulates the structural and functional properties of natural viruses (10). Over time, the LV system has evolved from two- and three-plasmid to the current four-plasmid system. This improvement enhances biosafety by reducing the risk of replication-competent PsV formation and preventing the risk of oncogene induction caused by the integration of long terminal repeats (LTRs) into the host genome (11, 12). The four-plasmid LV system has already been successfully employed to generate PsV-based QCMs for a range of various viruses, including SARS-CoV-2 and MERS-CoV (13, 14). Despite its success in other viral contexts, the application of the four-plasmid LV system for HIV-1 NAT-QCM production remains underexplored, necessitating further research.

In this study, using an improved four-plasmid LV system, we developed a novel HIV-1 PsV-based QCM for HIV-1 NAT. The homogeneity, stability, and matrix effects were evaluated. Furthermore, an external quality assessment (EQA) involving 60 laboratories was conducted to validate the accuracy and stability of the HIV-1 PsV-based QCM compared with those of inactivated HIV-1.

## MATERIALS AND METHODS

### Design and modification of transfer plasmids

To prevent generating replication-competent viruses, the four-plasmid LV system is designed by separating the essential components of HIV-1 into distinct plasmids: two packaging plasmids pMDLgag-pol RRE expressing Gag, Pol and Rev response element (RRE) of HIV-1, and pRSV-Rev expressing HIV-1 Rev; an envelope plasmid pMD2.G providing vesicular stomatitis virus-glycoprotein (VSV-G); and a transfer plasmid pLL3.7 containing truncated LTRs, Ψ packaging signal, RRE and a central polypurine tract/central termination sequence (cPPT/CTS) element of HIV-1, as well as a transgene region and a green fluorescent protein (GFP) as reporter. Through the four-plasmid system, the sequence between the 5′ LTR and 3′ LTR carried by the transfer plasmid is efficiently packaged into PsV with viral capsids and viral envelope (15). The four-plasmid LV system was purchased from Biomed (Beijing, China). Restriction enzymes were purchased from New England Biolabs (Ipswich, MA, USA).

**Fig 1:**
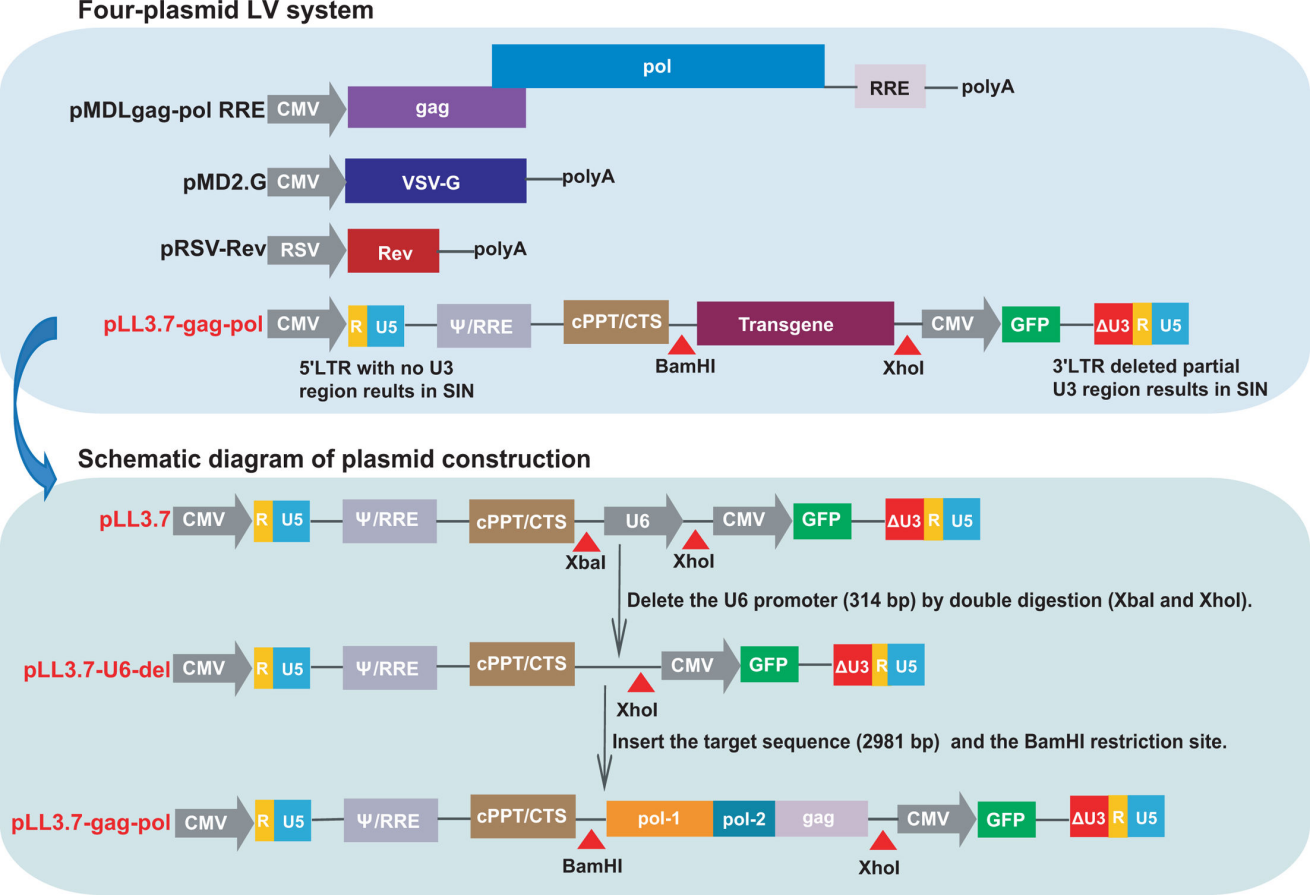
Schematic diagram of the modified four-plasmid LV system. SIN, self-inactivating.

Considering that the LV system is derived from the HIV-1 backbone, to reduce the risk of active replication when additional HIV-1 genes are inserted, we modified the four-plasmid LV system. Specifically, the U6 promoter (314 bp) in the transfer plasmid pLL3.7 was deleted using the restriction enzymes XbaI and XhoI to generate the transfer plasmid pLL3.7-U6-del, thereby preventing protein expression of the inserted gene (Fig. 1). For the development of the PsV-based HIV-1 NAT QCM, we designed a strategy to insert a conserved sequence of HIV-1 that is amplifiable by commercial HIV-1 NAT kits. A total of 884 genomic sequences of HIV-1 strains circulating in China were downloaded from the HIV Database (https://www.hiv.lanl.gov/) and aligned with the HXB2 reference strain to identify HIV-1 conserved regions. Excluding the conserved regions inherent to the transfer plasmid (e.g., LTRs, partial segment of pol), the residual sequence was selected as the target sequence, which included pol-1 segment (2,328–4562 bp), pol-2 segment (4,956–5,130 bp), and gag segment (1,280–1,850 bp). Subsequently, the target sequence was synthesized, verified by Sanger sequencing (Biomed), and seamless cloned into the transgene region of the transfer plasmid pLL3.7-U6-del between the BamHI and XhoI restriction sites, resulting in the construction of the transfer plasmid pLL3.7-gag-pol. Both transfer plasmids of pLL3.7-U6-del and pLL3.7-gag-pol were validated by 1% DNA agarose gel electrophoresis.

### Production and titer of HIV-1 PsV

HEK‐293T cells were cultured in Dulbecco’s modified essential medium (DMEM; Gibco) supplemented with 10% fetal bovine serum (FBS; Gibco) at 37°C with 5% CO_2_. A total of 1 × 10^7^ cells were seeded and allowed to adhere for 24 h in a T75 culture flask. Subsequently, four plasmids pLL3.7-gag-pol, pRSV-Rev, pMDLgag-pol RRE, and pMD2.G were co-transfected into the HEK-293T cells at 8, 4, 4, 4 µg, respectively, using Lipofectamine 2000 (Thermo Fisher Scientific) according to the manufacturer’s instructions. After transfection, the nuclei were stained with DAPI as previously described (16), and GFP fluorescence was observed and recorded using a fluorescence microscope. The concentration of p24 antigen in the supernatant was measured at days 1–5 post-transfection using the HIV-1 p24 Antigen ELISA Kit (Hebei Medical University Biological Products, Hebei, China), following the previously described protocol (17). To determine the titre of packaged HIV-1 PsV, the supernatant was serially diluted 10-fold and added to HEK-293T cells seeded in 96-well plates. The number of GFP-positive and GFP-negative wells was recorded for each dilution, and the TCID_50_ was calculated using the Reed-Muench method (18).

### Transmission electron microscopy (TEM)

The morphology of the HIV-1 PsV particles was observed using TEM. Briefly, the PsV-containing supernatant was ultracentrifuged at 100,000 × *g* for 2.5 h at 4°C over a 25% sucrose cushion using a Himac CP-NX ultracentrifuge. The pelleted PsV particles were subsequently resuspended in 0.01M phosphate-buffered saline (PBS; pH 7.4). The resuspended particles were then adsorbed onto TEM grids, negatively stained with 1% phosphotungstic acid (pH 6.8), and observed under a TEM (FEI Tecnai 12, USA).

### Nucleic acid extraction, PCR, and RT-PCR

To confirm the insertion of the target sequence into the HIV-1 PsV, a primer pair was designed to flank the transgene region of the transfer plasmid. The information on primers (F1 and R1) is shown in Table 1. The supernatant containing PsV and the PsV-infected HEK-293T cells were processed for nucleic acid extraction using a QIAamp Viral RNA/DNA Mini Kit (QIAGEN, Hilden, Germany). Subsequently, RT-PCR and PCR (Takara, Japan) were performed to amplify the target sequence from the supernatant and the infected cells, respectively.

**TABLE 1:** Primers and probe used in this study

Primer/probe	Sequence (5′–3′)	Test objective
F1	TGCAGGGGAAAGAATAGTAGAC	The PCR or RT-PCR for identifying the target sequence packaged into the HIV-1 PsV.
R1	AGCTCTGCTTATATAGACCT	
F2	GGGAGCTCTCTGGCTAACTA	RT-dPCR for the quantification of HIV-1 PsV.
R2	TTACCAGAGTCACACAACAGAC	
Probe2	TGCCTTGAGTGCTTCAAGTAGTGTGTG	

### Evaluation of infectivity of HIV-1 PsV

To ensure the infectivity of HIV-1 PsV, HEK-293T cells were infected with the PsV. After 4 h, the cells were thoroughly washed using PBS, and the medium was replaced with fresh DMEM, followed by incubation for 48 h. The supernatant was then collected and co-cultured with newly seeded HEK-293T cells. GFP fluorescence was observed over the next 48 h.

### Preparation of three types of QCMs

To prepare HIV-1 PsV-based QCM, the supernatant containing HIV-1 PsV was collected and centrifuged at 2,000 rpm for 10 min at 4°C to remove cell debris and was filtered through a 0.45 µm filter. For comparative analysis of different QCMs, MS2 and inactivated HIV-1 were also prepared. A conserved sequence of 622 bp encompassing LTR and gag was synthesized, ligated into the MS2-vector (pET-MS2), and transformed into competent *Escherichia coli* cells. Products were harvested, sonicated, purified, synthesized, and packaged by Sangon Biotech (Shanghai, China). The HIV-1 strain of subtype B stored in the HIV reference laboratory of the National Center for AIDS/STD Control and Prevention (NCAIDS/STD) was inactivated by heating at 56°C for 30 mins. To mimic clinical plasma samples, HIV-1 negative plasma was utilized as the dilution matrix for QCMs. Negative plasma was filtered through a 0.22 µm filter. HIV-1 PsV, MS2, and inactivated HIV-1 were diluted with negative plasma to prepare high and low concentrations QCMs. QCMs were assembled into 1 mL per vial and immediately stored at −80°C.

### Reverse transcription digital PCR (RT-dPCR)

To precisely quantify the QCMs, amplification primers and a probe were designed targeting a conserved region of LTR. The information on primers and probe (F2 and R2, and Probe2) is shown in Table 1. A RT-dPCR detection method was subsequently established (19–21).

The RT-dPCR was performed using the QIAcuity One Instrument and the QIAcuity OneStep Advanced Probe Kit (QIAGEN, Hilden, Germany). The RT-dPCR reaction system was as follows: 10 µL of 4 × OneStep Advanced Probe Master Mix, 0.4 µM of each primer, 0.2 µM of probe, 0.4 µL of 100 × OneStep Advanced RT Mix, 5 µL of GC enhancer, 10 µL of template, and ddH_2_O to make up a final volume of 40 µL. The reaction conditions were as follows: reverse transcription at 50°C for 40 min; pre-denaturation at 95°C for 2 min; followed by 40 cycles of 95°C for 5 s, 55°C for 15 s, and 72°C for 20 s. The dynamic range of the RT-dPCR assay was evaluated using a series of fivefold serial dilutions (1:1,000, 1:5,000, 1:25,000, and 1:125,000) of the PsV supernatant, which was collected 48 h post-transfection. The resulting standard regression equation was determined to be *y* = −0.9164*x* + 6.643, with a correlation coefficient (*R*) of 0.999 (Fig. S1). All RT-dPCR results are expressed in log10 copies/mL.

### Matrix effects, homogeneity, and stability of QCMs

In accordance with the guideline (22), the matrix effects were evaluated using 20 HIV-1 positive plasma samples and four PsV-based QCMs diluted in HIV-1 negative plasma at concentrations of 10^6^, 10^5^, 10^4^, and 10^3^ copies/mL. The analysis was performed using HIV-1 NAT quantification kits from Livzon (Zhuhai, China) and Wantai (Beijing, China). Each sample was tested in triplicate, and the results are reported as the mean log10 copies/mL values. Linear regression analysis was conducted to assess the matrix effects, with the mean values obtained from the Livzon method plotted on the *X*-axis and those from the Wantai method on the *Y*-axis. The HIV-1 positive plasma utilized in this study was from stored samples in the HIV reference laboratory of NCAIDS/STD.

For the homogeneity analysis, in accordance with the guidance (23, 24), 10 units of each QCM were assessed for two replicates using RT-dPCR. The short-term stability and freeze-thaw stability were evaluated in six samples per experiment using RT-dPCR. For the short-term stability test, the QCMs were stored at −20°C, 4°C, and room temperature 25°C for days 0, 1, 2, 3, 5, and 7, and the QCMs were subsequently quantitated. For freeze-thaw stability, the QCMs stored at −80°C were melted at room temperature 25°C and then frozen again at −80°C (one to five times).

### EQA application with HIV-1 PsV and inactivated HIV-1

To evaluate the HIV-1 PsV-based QCM prepared in this study across various testing platforms, 12 commercial HIV-1 NAT quantification kits, which are widely employed in HIV-1 NAT laboratories across China, were tested.

To further explore the applicability of HIV-1 PsV-based QCM in EQA, we used high-concentration HIV-1 PsV-based QCM and inactivated HIV-1 QCM to form the EQA panel. The panels were distributed to 60 laboratories, including 30 laboratories using the Roche HIV-1 quantitative NAT testing kits (Switzerland) and 30 laboratories using the Livzon HIV-1 quantitative NAT testing kits (Zhuhai, China) as study participants. Each laboratory received two panels, with one panel tested during the first half of the year and the other during the second half. The EQA panels were transported via a cold chain system and stored at −80°C until testing. All participating laboratories were blinded to the sample details and required to report their results within 4–6 weeks in accordance with EQA guidelines (25).

### Statistical analysis

The data were analyzed using GraphPad Prism v8.0 (San Diego, USA) and SPSS v26.0 (IBM Corp., USA). Matrix effects were analyzed through linear regression (22). Homogeneity was evaluated using one-way ANOVA, while stability was assessed using Student’s *t*-test (23, 24). The EQA results were evaluated using a paired *t*-test.

## RESULTS

### HIV-1 PsV was produced by the modified four-plasmid LV system

**Fig 2:**
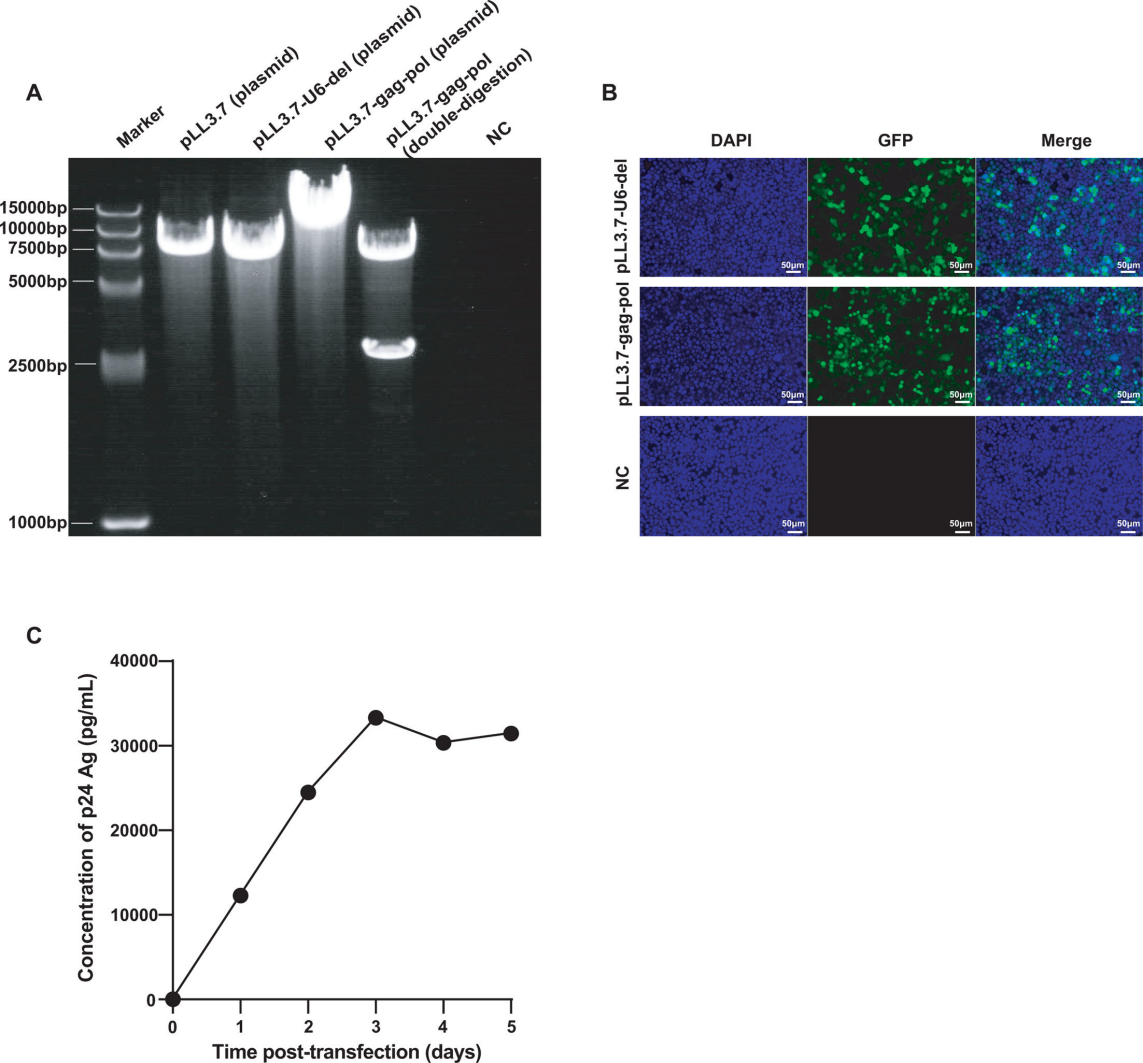
Transfer plasmid construction and HIV-1 PsV production. (**A**) Gel electrophoresis image from left to right: Marker, plasmid pLL3.7 (7,650 bp), pLL3.7-U6-del (7,336 bp), pLL3.7-gag-pol (10,084 bp), double-digested pLL3.7-gag-pol (7,103 and 2,981 bp), and negative control (NC). (**B**) Fluorescence microscopy of HEK-293T cells transduced with LV systems using different plasmids (pLL3.7-U6-del and pLL3.7-gag-pol) for 48 h; un-transfected HEK-293T cells served as NC. (**C**) The concentration of the p24 antigen in the PsV supernatant from days 1 to 5 post-transfection, with time on the *X*-axis and p24 Ag concentration on the *Y*-axis.

The electrophoresis results demonstrated that the band position of the plasmid pLL3.7 and the plasmid pLL3.7-U6-del were consistent with the expected result of 7,650 and 7,336 bp, indicating that the U6 promoter was successfully deleted from the plasmid pLL3.7. Furthermore, the electrophoretic analysis on the digested products of plasmid pLL3.7-gag-pol (10,084 bp) by BamHI and XhoI showed bands at 7,103 and 2,981 bp, confirming the successful insertion of the target sequence (Fig. 2A).

GFP fluorescence was observed at 48 h post-transfection, while no fluorescence was detected in un-transfected cells (Fig. 2B), confirming the successful transfection of the transfer plasmid pLL3.7-gag-pol. The concentration of the HIV-1 p24 antigen in the supernatant increased rapidly from days 1 to 3 and reached a plateau at days 4 and 5 (Fig. 2C). Additionally, the harvested supernatant exhibited a PsV titer of 1 × 10⁶ TCID_50_/mL, indicating that the packaged HIV-1 PsV was capable of infecting HEK‐293T cells. Both the p24 Ag and TCID_50_ results demonstrated the successful packaging of HIV-1 PsV. Based on RT-dPCR results of serial dilutions of the PsV supernatant, the average concentration of the PsV supernatant was estimated to be 1.88 × 10^9^ copies/mL through back-calculation using the dilution factors.

### HIV-1 PsV has a virus-like structure but is non-infectious

**Fig 3:**
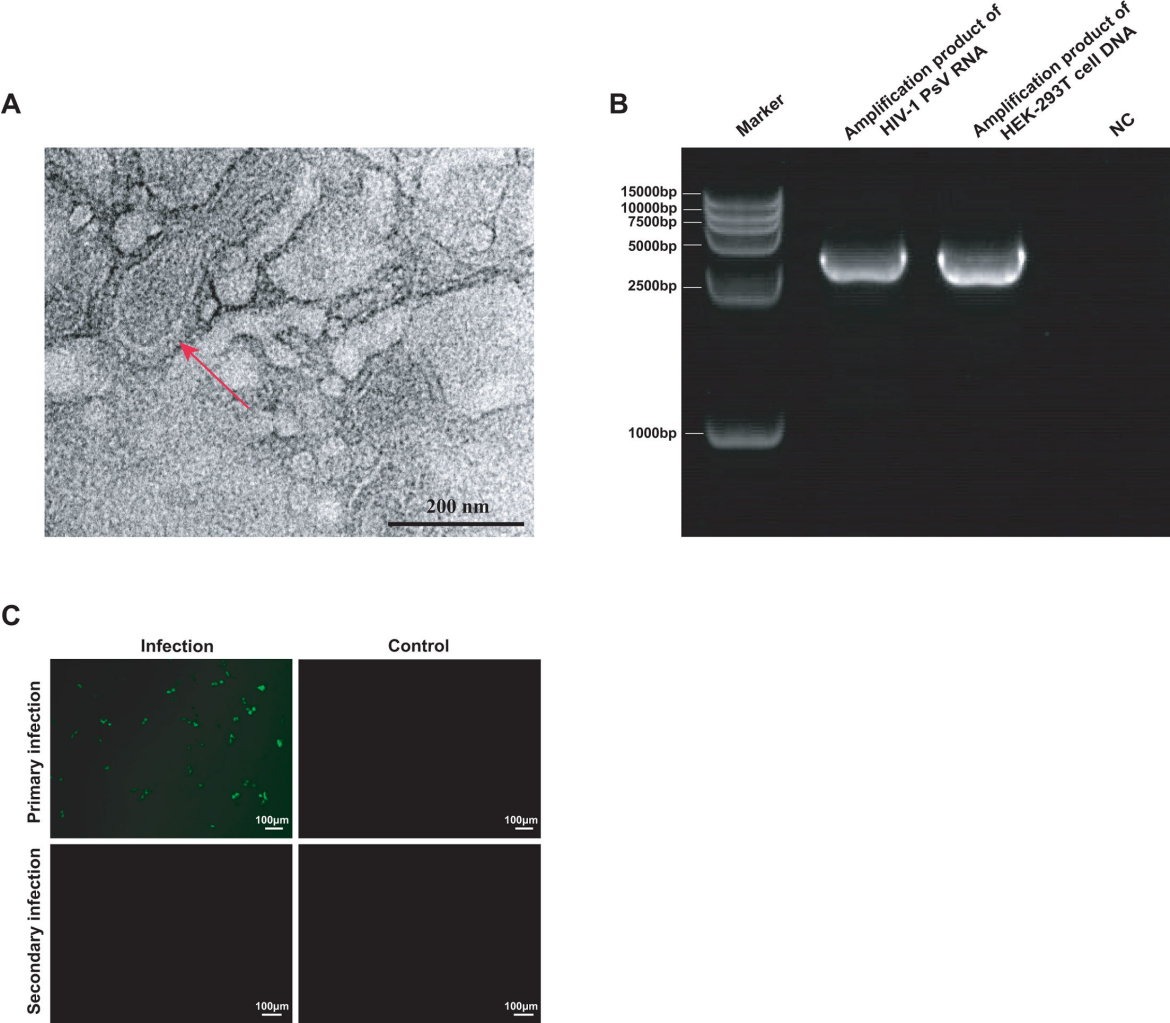
Identification and characterization of HIV-1 PsV. (**A**) Representative TEM image of HIV-1 PsV particles as indicated by red arrows. (**B**) Gel electrophoresis image, from left to right: Marker, the amplification product of HIV-1 PsV RNA (3,622 bp), the amplification product of HEK‐293T cell DNA (3,622 bp), and negative control (NC). (**C**) Fluorescence microscopy analysis of HEK-293T cells infected with HIV-1 PsV. Primary infection: HEK-293T cells were infected with the HIV-1 PsV; secondary infection: HEK-293T cells were infected with the culture supernatant of primary infection; uninfected HEK-293T cells served as NC.

The TEM results observed that the produced HIV-1 PsV particles had a round shape with membrane structure, and the particle size was around 100 nM (Fig. 3A). The electrophoresis results showed that the length of amplification products of HIV-1 PsV and HEK-293T cells was consistent with the expected result of 3,622 bp (Fig. 3B). Following a 48-h blind passage of the second-generation culture supernatant, no GFP fluorescence was detected in HEK‐293T cells (Fig. 3C), confirming that HIV-1 PsV is capable of only a single round of infection and no infectivity.

### HIV-1 PsV-based QCM shows no matrix effects and exhibits good homogeneity

**Fig 4:**
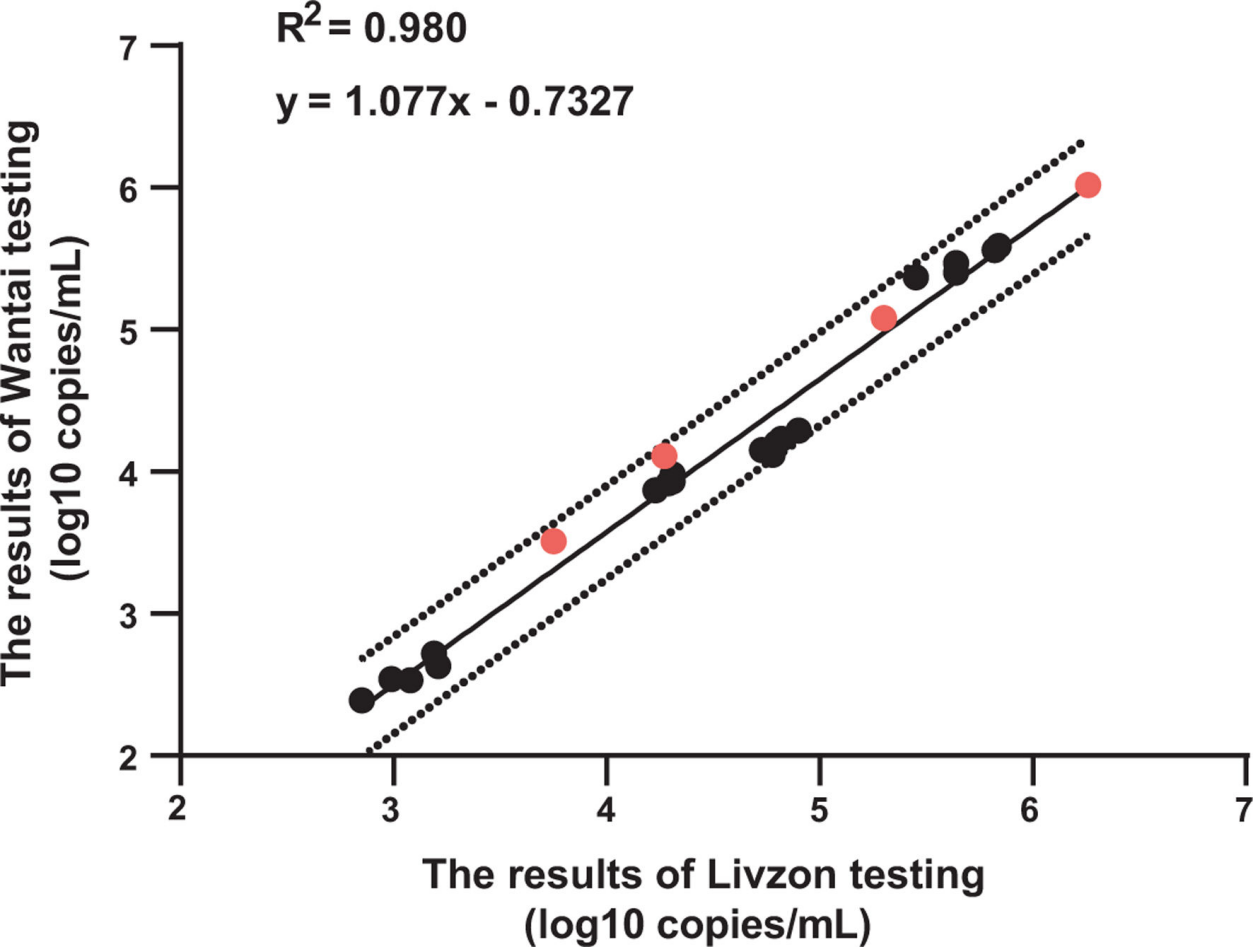
Matrix effects analysis of HIV-1 PsV-based QCM. The *X*-axis represents the results of Livzon testing; the *Y*-axis represents the results of Wantai testing. The solid line in the figure represents the regression curve derived from measurements of HIV-1 positive plasma and HIV-1 PsV-based QCM, while the dashed lines denote the 95% confidence intervals of the predicted values (Y). The black circles represent HIV-1 positive plasma, and the red circles represent the PsV-based QCM diluted in a negative plasma matrix.

The measured values of HIV-1 PsV-based QCM using the Livzon and Wantai assays all fell within the 95% confidence interval of the regression line. The matrix effect results demonstrated that HIV-1 negative plasma, used as a matrix medium, did not affect the quantitation (Fig. 4).

To evaluate the homogeneity of HIV-1 PsV-based QCM, we compared the performance of HIV-1 PsV-based QCM with the QCMs prepared by MS2 and inactivated HIV-1. Homogeneity analysis showed a critical *F* value of *F*_0.05(9, 10)_ = 3.02. The calculated *F* values for all three groups were below this threshold with *P* > 0.05, demonstrating no statistically significant differences (Table 2). The above results demonstrate that all three types of QCMs exhibit satisfactory homogeneity, which ensures that the quantitative results are valid with any QCM vial regardless of the filling order.

**TABLE 2:** Homogeneity analysis of three types of QCMs^*a*^

Sample no.	HIV-1 PsV				MS2				Inactivated HIV-1			
	H-1	H-2	L-1	L-1	H-1	H-2	L-1	L-1	H-1	H-2	L-1	L-1
1	5.10	5.02	3.23	3.48	5.27	5.31	3.53	3.40	5.12	5.12	3.26	2.36
2	5.02	5.00	3.26	3.51	5.25	5.25	3.22	3.11	5.11	5.19	3.27	3.36
3	5.00	5.06	3.27	3.15	5.32	5.30	3.29	3.47	5.18	5.10	3.30	3.84
4	5.07	5.09	3.36	3.36	5.25	5.27	3.52	2.78	5.13	5.12	3.32	3.18
5	5.00	5.09	3.48	3.39	5.31	5.26	3.02	3.37	5.14	5.05	2.95	2.65
6	5.02	5.07	3.26	3.40	5.25	5.27	3.33	3.52	5.12	5.11	3.06	3.59
7	5.02	5.03	3.25	3.33	5.25	5.29	3.42	3.19	5.12	5.12	3.43	2.93
8	5.01	5.03	3.32	3.19	5.28	5.17	3.45	3.27	5.16	5.13	3.09	2.95
9	4.95	4.96	3.47	3.40	5.30	5.32	3.36	3.24	5.14	5.13	3.43	3.26
10	5.08	5.02	3.40	3.40	5.25	5.27	3.49	3.25	5.14	5.16	3.21	2.96
Overall mean	5.03		3.35		5.27		3.31		5.13		3.17	
*F* value	0.27		0.47		0.02		1.53		0.95		0.72	
*P* value	0.61		0.63		0.90		0.23		0.34		0.41	

^*a*^ H represents the QCM at a high concentration; L represents the QCM at a low concentration. Assay results were converted to log10 copies/mL.

### Comparison of stability of three types of QCMs

**Fig 5:**
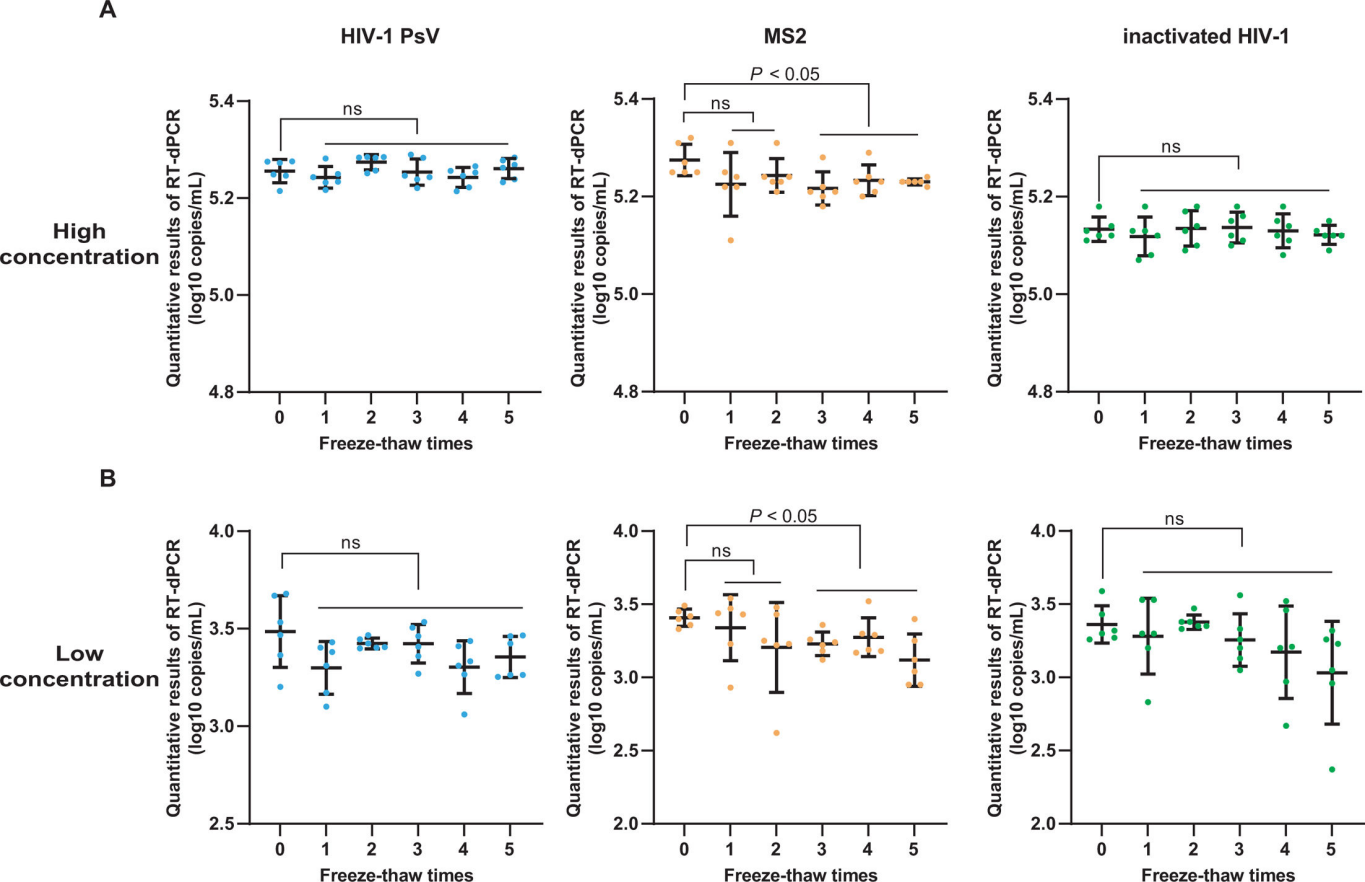
Freeze-thaw stability analysis of three types of QCMs. The *X*-axis represents the freeze-thaw times, the *Y*-axis represents the quantitative results of RT-dPCR (log10 copies/mL). (**A**) Results of high-concentration QCMs. (**B**) Results of low-concentration QCMs (ns: *P* > 0.05).

For the freeze-thaw stability, at both high and low concentrations, the QCMs prepared with HIV-1 PsV or inactivated HIV-1 maintained stability even after undergoing five freeze-thaw times. In contrast, the stability of QCM prepared with MS2 was significantly compromised after two freeze-thaw times (Fig. 5).

**Fig 6:**
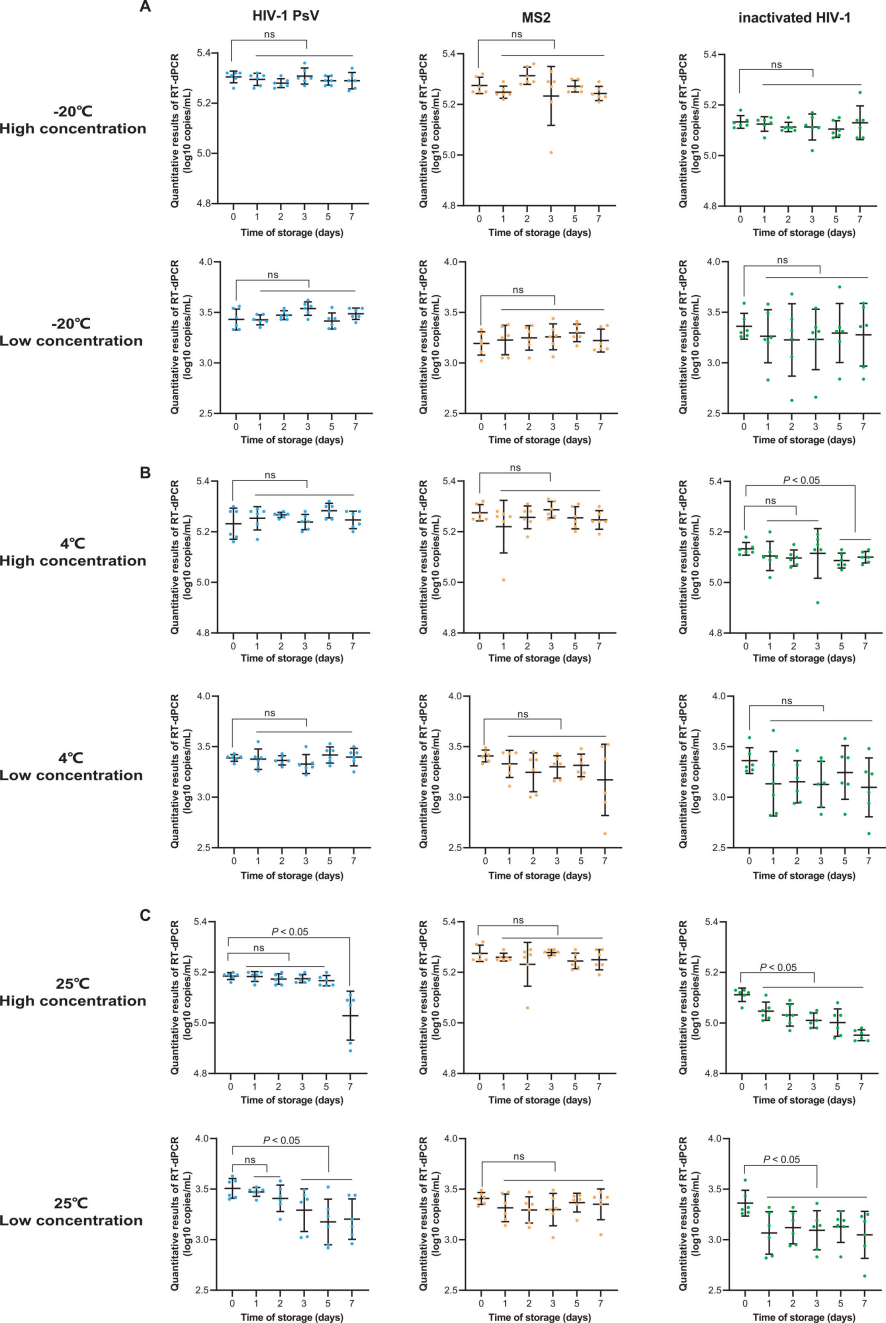
Short-term stability analysis of three types of QCMs. The *X*-axis represents the time of storage, the *Y*-axis represents the quantitative results of RT-dPCR (log10 copies/mL). (**A–C**) Results of high- and low-concentration QCMs at −20°C, 4°C, and room temperature 25°C, respectively (ns: *P* > 0.05).

The short-term stability results are shown in Fig. 6. At −20°C, all three types of QCMs remained stable for 7 days (Fig. 6A). At 4°C, with the exception of the high-concentration inactivated HIV-1 QCM, which retained stability for only 3 days, all other QCMs remained stable for 7 days (Fig. 6B). At room temperature of 25°C, both the high and low concentrations of MS2 QCM retained stability for 7 days. The high and low concentrations of HIV-1 PsV-based QCM retained stability for 5 and 2 days, respectively (Fig. 6C). In contrast, the stability of both high and low concentrations of inactivated HIV-1 QCM was significantly decreased after 1 day at 25°C.

### EQA application of QCMs prepared with HIV-1 PsV and inactivated HIV-1

All 12 commercial HIV-1 NAT quantification kits, including 2 imported reagents and 10 domestic reagents, could all successfully detect the HIV-1 PsV-based QCMs, and the results of 12 reagents were all within the mean ± 0.5 log value. The amplification target region information of the 12 reagents is presented in Table 3.

**TABLE 3:** Targets of 12 commercial HIV-1 NAT quantification kits that detected HIV-1 PsV-based QCM

Imported or domestic	Manufacturer	Targets
Imported	Roche	gag + LTR
	Hologic	pol + LTR
Domestic	Revvity	gag + pol
	Amplly	gag + LTR
	Da an	gag + LTR
	Geneway	gag + LTR
	Northeast Pharm	gag + LTR
	Livzon	pol + LTR
	Sansure	pol + LTR
	Wantai	gag + pol + LTR
	Rendu	pol
	Bio-resource	gag

Within the allotted time, we received the results reported by all 60 laboratories, achieving a 100% return rate. The CV values of 30 laboratories using Roche reagent for the HIV-1 PsV and inactivated HIV-1 EQA samples were 3.55% and 1.79%, respectively. Similarly, the CV values of 30 laboratories using Livzon reagent for the HIV-1 PsV and inactivated HIV-1 EQA samples were 3.73% and 1.89%, respectively. Both QCMs exhibited CVs below 5%. Furthermore, when we compared the QCM test results from the first and second halves of the year in the same laboratory, the paired *t*-test results showed that there was no statistically significant difference in the test results between the two periods for both QCMs prepared with the HIV-1 PsV and inactivated HIV-1 (*P* > 0.05) (Table 4), demonstrating that both types of QCMs exhibit good stability. Moreover, the same results were obtained regardless of whether Roche or Livzon reagents were used (Table 4).

**TABLE 4:** The EQA results of 60 laboratories using HIV-1 PsV-based QCM and inactivated HIV-1 QCM^*a*^

Laboratory no.	Roche (inactivated HIV-1)		Roche (HIV-1 PsV)		Laboratory no.	Livzon (inactivated HIV-1)		Livzon (HIV-1 PsV)	
	First^*b*^	Second^*c*^	First	Second		First	Second	First	Second
1	5.62	5.70	5.39	5.39	1	5.76	5.53	4.90	4.79
2	5.85	5.86	5.45	5.64	2	5.71	5.67	4.97	4.90
3	5.42	5.86	4.91	5.29	3	5.62	5.65	4.85	4.91
4	5.82	5.90	5.22	5.69	4	5.53	5.59	4.68	4.84
5	5.86	5.76	5.26	5.23	5	5.66	5.68	4.77	4.79
6	5.85	5.93	5.53	5.45	6	5.57	5.59	4.77	4.82
7	5.87	5.88	5.37	5.29	7	5.82	5.65	4.91	4.90
8	5.80	5.86	5.55	5.46	8	5.65	5.68	4.85	4.85
9	5.61	5.86	5.20	5.03	9	5.68	5.51	4.99	4.97
10	5.74	5.74	5.33	5.35	10	5.76	5.68	4.97	4.97
11	5.96	6.01	5.49	5.46	11	5.73	5.70	4.82	4.79
12	5.82	5.60	5.19	5.40	12	5.73	5.70	5.10	5.10
13	5.78	5.66	5.33	5.38	13	5.57	5.70	4.74	4.73
14	5.80	5.97	5.42	5.46	14	5.50	5.83	4.76	4.80
15	5.76	5.30	5.17	3.84	15	5.60	5.65	4.46	4.56
16	5.76	5.30	5.07	5.03	16	5.52	5.05	4.63	4.73
17	5.83	5.79	5.35	5.18	17	5.74	5.62	4.89	5.64
18	5.73	5.96	4.85	5.42	18	5.71	5.77	4.17	4.34
19	5.94	5.73	5.37	5.54	19	5.54	5.59	4.63	4.79
20	5.80	5.83	5.25	5.31	20	5.54	5.52	4.80	4.89
21	5.99	5.76	5.41	5.39	21	5.60	5.59	4.83	4.82
22	5.93	5.91	5.30	5.34	22	5.55	5.50	4.77	4.61
23	5.85	5.86	5.25	5.28	23	5.70	5.65	4.96	4.96
24	5.93	5.73	5.27	5.34	24	5.40	5.47	4.63	4.76
25	5.69	5.65	5.13	5.26	25	5.68	5.46	5.00	4.83
26	5.67	5.87	5.21	5.38	26	5.63	5.60	4.89	4.95
27	5.76	5.79	5.17	4.21	27	5.72	5.82	4.77	5.08
28	5.97	5.19	5.43	5.21	28	5.55	5.60	4.69	4.97
29	5.72	5.73	5.36	5.28	29	5.37	5.43	4.57	4.69
30	5.92	5.90	5.38	5.46	30	5.62	5.57	4.80	4.71
Mean	5.79		5.32		Mean	5.61		4.82	
CV	1.79		3.55		CV	1.89		3.73	
*t *value	0.90		0.28		*t *value	0.78		−1.25	
*P* value	0.37		0.78		*P* value	0.44		0.22	

^*a*^ Assay results were converted to log10 copies/mL.

^*b*^ EQA results for the first half of the year.

^*c*^ EQA results for the second half of the year.

## DISCUSSION

In this study, we developed a novel HIV-1 NAT QCM, which is safe, stable, and is suitable for use as a QCM in the EQA program.

Biosafety of the QCMs for infectious pathogens is of paramount importance, while clinical samples and inactivated viruses that are commonly used for QCMs pose potential biosafety risks. PsV recombined and expressed using the LV system are commonly used to prepare QCMs for infectious viruses. Previous studies found that compared with the two- and three-plasmid LV systems, the four-plasmid LV system offers the highest level of biosafety. First, the four-plasmid system separates the *rev* gene from the packaging plasmid and expresses it on an independent plasmid while deleting the *tat* gene (26). Additionally, a portion of the promoter region in the 3′ LTR (U3 region) of the transfer plasmid was removed, rendering it replication-deficient and placing it in a “self-inactivating (SIN)” state (27). Also, the LTR promoter in the transfer plasmid was replaced with a heterologous promoter (28), eliminating the need for *tat*-mediated transcriptional enhancement or auxiliary *tat* protein expression. In this study, to further enhance the biosafety of the HIV-1 NAT QCM, we introduced, for the first time, an improved four-plasmid system strategy that was employed to prepare HIV-1 PsV for HIV-1 NAT QCM. In this improved system, we prevented the protein expression of the inserted sequence by deleting the U6 promoter from the transfer plasmid pLL3.7. The lack of GFP fluorescence in the second-generation blind passage confirmed that HIV-1 PsV produced in this study is capable of only single-round infection, demonstrating its biosafety.

In the four-plasmid LV system we used, the transfer plasmid pLL3.7 also included the cPPT/CTS sequence of the HIV-1 *pol* gene (29–31), which plays a critical role in the nuclear import of viral DNA and enhances the infection of non-dividing cells, thereby improving the transduction efficiency and titer of the LV. Therefore, within 2 days post-transfection, we could harvest HIV-1 PsV at a high concentration up to 1.88 × 10⁹ copies/mL. And high-concentration PsV can be produced in large quantities, facilitating long-term storage and meeting diverse research needs. Furthermore, HIV-1 PsV particles exhibit natural virus-like characteristics, including a diameter of approximately 100 nM and a spicule-like membrane structure (32, 33), which allow them to effectively simulate real clinical samples during nucleic acid extraction and detection, ensuring comprehensive quality control throughout the entire process. Previous studies have demonstrated the good stability of PsVs of SARS-CoV-2 and MERS-CoV packed by the four-plasmid LV system (13, 14). Furthermore, in our study, HIV-1 PsV exhibited better freeze-thaw stability than MS2 and superior short-term stability compared to inactivated HIV-1. Therefore, HIV-1 PsV produced in this study outperformed inactivated HIV-1 and MS2 in stability and is more suitable for use as a NAT QCM.

In this study, we aimed to develop a universal HIV-1 NAT QCM compatible with multiple commercial reagents. By analyzing 884 HIV-1 genomic sequences from China, we designed a target sequence with a length of 2,981 bp, encompassing conserved regions of HIV-1. The LV system can package external genes up to 8 kb length (34), which meets actual requirements. In contrast, the MS2 vector can only package external genes of approximately 2 kb (35, 36). Previous research has shown that due to the limited insertion sequence length of MS2, if different gene fragments are inserted into multiple MS2-vectors separately and these distinct MS2 are mixed as QCMs, significant variations in the quantified concentrations of different MS2 can occur (37). This is because the packaging efficiencies of individual gene fragments differ, leading to disparities even when mixed at the same ratio. As previous literature demonstrated, the nucleic acid extraction and target sequence amplification can be impacted by the efficiency of membrane lysis, virion size, and nucleic acid fragment size. The PsV employed in our QCM structurally recapitulates native virions through their Gag-derived protein shell, VSV-G lipid bilayer envelope, and viral RNA length (~7.4 kb vs HIV-1 ~9.7 kb). The design that mimics natural viruses enables the PsV to monitor two critical aspects of HIV-1 NAT on clinical samples: lysis of natural virions and amplification of viral RNA that has certain length and secondary structure. By mimicking both viral structure and nucleic acid complexity, HIV-1 PsV-based QCM provides superior simulation of NAT workflow.

HIV-1 PsV-based QCM developed exhibited significant application value in EQA. In this study, we incorporated the conserved regions of the HIV-1 genome into PsV particles. The resulting HIV-1 PsV-based QCM demonstrated broad compatibility, ensuring its utility across various platforms for both internal quality control and nationwide EQA programs. To assess its suitability for EQA, the HIV-1 PsV-based QCMs were distributed to 60 HIV-1 NAT laboratories from provincial-, prefecture-, and county-level hospitals and CDCs, utilizing either the imported Roche reagent or domestic Livzon reagent. Additionally, when we compared the QCM test results from the first and second halves of the year in the same laboratory, the results demonstrated that the stability assessment results of HIV-1 PsV and inactivated HIV-1 in the same laboratory are consistent.

In summary, to develop a novel HIV-1 NAT QCM, we have successfully established an HIV-1 PsV production platform using an improved four-plasmid LV system. This novel QCM prepared from HIV-1 PsV is non-infectious, and its transportation and storage do not require special biosafety containment measures. In addition, the novel QCM mimics natural viruses and can perform quality control throughout the entire NAT process, and it can be produced in large quantities, effectively reducing costs and showing great application potential. Our research indicates that these characteristics make this novel QCM suitable for EQA. The improved LV system we developed can also be utilized in the future to generate QCMs tailored to specific HIV-1 strains or newly identified variants. Additionally, it can be employed to insert viral sequences containing drug resistance sites, thereby developing QCMs for HIV genotypic drug resistance testing. This will provide robust support for quality control in laboratories conducting HIV-1 NAT and genotypic drug resistance testing.

## SUPPLEMENTAL MATERIAL

Fig. S1 spectrum.00269-25-s0001.pdf Linearity analysis of RT-dPCR detection method using serial dilutions of HIV-1 PsV.

ASM does not own the copyrights to Supplemental Material that may be linked to, or accessed through, an article. The authors have granted ASM a non-exclusive, world-wide license to publish the Supplemental Material files. Please contact the corresponding author directly for reuse.
