# Supplementary figures and images for: Development of a novel pseudovirus-based quality control material for HIV-1 nucleic acid testing and its application in external quality assessment
Source: Microbiol Spectr. 2025 Jun 10;13(7):e00269-25. doi: 10.1128/spectrum.00269-25 (PMC12210937; doi:10.1128/spectrum.00269-25)

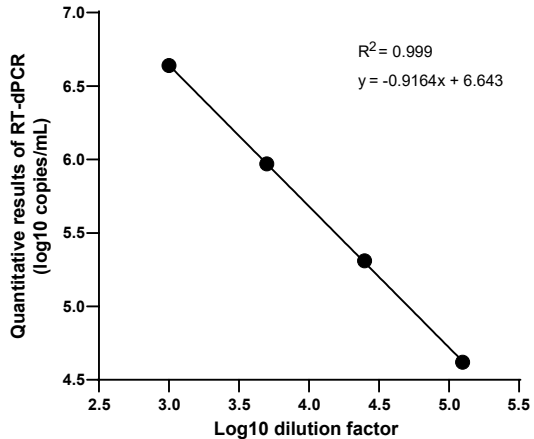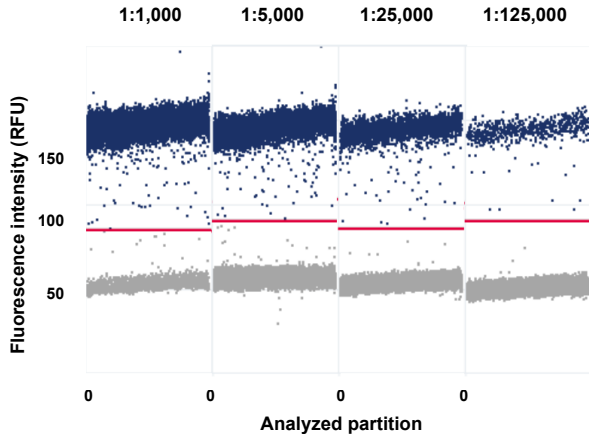

Supplement: Fig. S1 — Linearity analysis of RT-dPCR detection method using serial dilutions of HIV-1 PsV. [file spectrum.00269-25-s0001.pdf]
